# Clusters of Survivors of COVID-19 Associated Acute Respiratory Failure According to Response to Exercise

**DOI:** 10.3390/ijerph182211868

**Published:** 2021-11-12

**Authors:** Michele Vitacca, Mara Paneroni, Alberto Malovini, Annalisa Carlucci, Chiara Binda, Vincenzo Sanci, Nicolino Ambrosino

**Affiliations:** 1 Respiratory Rehabilitation of the Institute of Lumezzane, Istituti Clinici Scientifici Maugeri IRCCS, 25065 Lumezzane, Italy; mara.paneroni@icsmaugeri.it; 2 Laboratory of Informatics and Systems Engineering for Clinical Research of the Institute of Pavia, Istituti Clinici Scientifici Maugeri IRCCS, 27100 Pavia, Italy; alberto.malovini@icsmaugeri.it; 3 Respiratory Rehabilitation of the Institute of Pavia, Istituti Clinici Scientifici Maugeri IRCCS, 27100 Pavia, Italy; annalisa.carlucci@icsmaugeri.it (A.C.); c.binda3@studenti.uninsubria.it (C.B.); vincenzo.sanci01@universitadipavia.it (V.S.); 4 Department of Medicine and Surgery, Università Insubria, 21100 Varese, Italy; 5 Respiratory Rehabilitation of the Institute of Montescano, Istituti Clinici Scientifici Maugeri IRCCS, 27040 Montescano, Italy; nico.ambrosino@gmail.com

**Keywords:** acute respiratory failure, dyspnoea, exercise test, exercise tolerance, fatigue, pulmonary rehabilitation, six-minute walking test

## Abstract

COVID-19 survivors are associated with acute respiratory failure (ARF) and show a high prevalence of impairment in physical performance. The present studied aimed to assess whether we may cluster these individuals according to an exercise test. The presented study is a retrospective analysis of 154 survivors who were admitted to two hospitals of Istituti Clinici Scientifici Maugeri network, Italy. Clinical characteristics, walked distance, heart rate (HR), pulse oximetry (SpO_2_), dyspnoea, and leg fatigue (Borg scale: Borg-D and Borg-F, respectively) while performing the six-minute walking test (6MWT) were entered into unsupervised clustering analysis. Multivariate linear regression identified variables that were informative for the set of variables used for cluster definition. Cluster 1 (C1: 86.4% of participants) and Cluster 2 (C2: 13.6%) were identified. Compared to C1, the individuals in C2 were significantly older, showed significantly higher increase in fatigue and in dyspnoea, greater reduction in SpO_2_, and a lower HR_peak_ during the test. The need of walking aids, time from admission to acute care hospitals, age, body mass index, endotracheal intubation, baseline HR and baseline Borg-D, and exercise-induced SpO_2_ *change* were significantly associated with the variables that were used for cluster definition. Different characteristics and physiological parameters during the 6MWT characterise survivors of COVID-19-associated ARF. These results may help in the management of the long-term effects of the disease.

## 1. Introduction

The SARS-coronavirus-2 disease 19 (SARS-CoV-2) pandemic has had dramatic effects throughout the world, with more than two hundred, thirty million people infected and more than four and half million casualties worldwide, requiring comprehensive management strategies in the acute as well as in the post-acute phases of the pandemic [1,2]. In addition to the consequences on lung function [3], a high prevalence of impairment in physical performance is reported in survivors, who may suffer from fatigue and/or muscle weakness, exercise-induced dyspnoea, sleep difficulties, and anxiety and/or depression up to 6 months after infection [4,5,6,7]. In addition, persistent exercise-induced desaturation (EID) was reported in up to 43% of survivors of acute respiratory failure (ARF) due to COVID-19 pneumonia [8], and these individuals, when normoxaemic at rest with EID, may show alterations in lung function, exercise capacity, and symptoms similar to individuals with interstitial lung diseases but that are more severe than those of individuals with chronic obstructive pulmonary disease (COPD) and EID [9].

We wondered whether these individuals might be characterized on the basis of physiological response to symptoms during an exercise test. Cluster analysis consists of methods to identify subgroups of data. By assigning items into the subgroups that are identified, clustering can help us to understand the characteristics of the patterns present in data. These techniques are commonly used in a wide range of areas, including medicine, psychology, market research, and bioinformatics [10].

Therefore, the aim of this study was to assess whether survivors of COVID-19 with associated ARF, and who were normoxaemic at rest, could be clustered according to exercise capacity and physiological and symptom response to an exercise test.

## 2. Materials and Methods

This retrospective case series study was approved by the Istituti Clinici Scientifici (ICS) Maugeri, IRCCS Ethics Committee (2440 CE). As a retrospective study, the individuals did not need to provide any specific written informed consent; however, at admission they gave informed consent for the scientific use of their clinical data in advance. As this was a retrospective analysis, the study was not registered.

### 2.1. Participants

The participants included individuals who were consecutively admitted between 15th April and 30th May 2020 to the Respiratory Units of ICS Maugeri, IRCCS hospitals of Lumezzane (Brescia) and Pavia—Italy, referral institutions for pulmonary rehabilitation, diagnosis, and care of post-acute and chronic conditions [11,12]. These individuals had been transferred from acute care hospitals where they had undergone invasive or non-invasive (NIV) ventilation, high flow oxygen therapy, and/or oxygen supplementation [13].

The inclusion criteria were negative Real Time PCR (RT-PCR) test for SARS-CoV-2 at the time of evaluation, ability to perform the six-minute walking test (6MWT) at discharge from hospital, and to be normoxaemic at rest. 

The exclusion criteria were the need of oxygen supplementation at the time of evaluation or the need of Long-Term Oxygen Therapy (LTOT), the presence of neurological or orthopaedic conditions (chronic or new onset), and inability or unwillingness to undergo evaluations.

### 2.2. Measurements

The following data were recorded from the ICS clinical data repository: demographics, anthropometrics, comorbidities, length of stay (LoS) in acute care and in our hospitals, history of endotracheal intubation (EI) and mechanical ventilation, NIV or oxygen supply in the acute care hospitals, presence of tracheostomy, need of walking aids, Motor Barthel index [14], and serological parameters (C-reactive protein [CRP], D-dimer, ferritin, albumin, haemoglobin) at admission.

With the appropriate safety procedures and while wearing appropriate personal protective equipment [15], the participants underwent arterial blood gases under room air at admission. 

Exercise tolerance was assessed by means of the 6MWT according to accepted standards [16]. The 6MWT is the most worldwide used and accepted field test to assess exercise capacity in different conditions and setting including post-COVID-19 and during rehab [5]. The walked distance was expressed as meters and as the percent of the reference values [17]. At the beginning and at the end of the walking cycle, subjective sensations of dyspnoea (Borg-D) and leg fatigue (Borg-F) were assessed by means of the modified Borg scale [18]. Peripheral oxygenation (SpO_2_) and heart rate (HR) were monitored using a pulse oximeter (VintusWalk, VYAIRE MEDICAL, INC, NY, USA)**.** Baseline and SpO_2 nadir_ and baseline and HR_peak_ were also recorded. Exercise-induced desaturation was defined as SpO_2 nadir_—baseline SpO_2_ > 4% during the 6MWT [19]. Heart rate and % of maximal predicted (220—age) was also recorded.

### 2.3. Statistical Analysis

Continuous and discrete numeric variable distribution was described as the median and interquartile range (25th, 75th percentiles, IQR) since most of them deviated from the normal distribution based on visual inspection of histograms. Categorical variable distribution was described as the absolute and relative frequency (%). Change in continuous and discrete numeric variables between baseline and end of the 6MWT were calculated as *end-exercise*–baseline values.

Unsupervised hierarchical agglomerative clustering analysis was performed using the Euclidean distance between the participants and the complete linkage function after data centring and scaling using the mean and standard deviation (SD) of each variable’s distribution. The information for the distance during the 6MWT, % predicted, HR *change* (HR_peak_—baseline HR), SpO_2_ *change* (SpO_2 nadir_—baseline SpO_2_), Borg-D *change* (*end-exercise* Borg-D—baseline Borg-D), and Borg-F *change* (*end-exercise* Borg-F—baseline Borg-F) were used for cluster identification on the overall dataset and included participants from both study centres. The optimal number of clusters was identified as the one guaranteeing the highest silhouette coefficient value by testing different numbers of clusters (from 1 to 20). 

The Spearman correlation coefficient *r* and corresponding 95% confidence interval (95% CI) were calculated to estimate the strength of the correlation between continuous and discrete numeric variables. The Wilcoxon rank sum test was applied to compare numeric variable distribution between groups. Pearson chi-square test with p-values computation using the Monte Carlo simulation (10,000 simulations) was applied to test for association between categorical variables.

Multivariate linear regression with stepwise forward features selection based on the Bayesian Information Criterion (BIC) was applied to identify the most informative subset of variables associated with the response variable of interest. Before multivariate linear regression fitting, missing values of numeric continuous and discrete variables with a missing data fraction ≤20% were imputed by the median value of the corresponding distribution and the missing values of the categorical variables by the most frequent value of the corresponding frequency distribution. Variables that were characterized by a missing data fraction >20% were not included in the analysis. A starting set of variables including the hospital centre and the baseline value of each response variable was forced to be included into the model. The set of potentially informative variables based on clinical knowledge was represented by LoS in acute hospitals (days), LoS in study centres (days), time from acute hospital admission (days), age (years), body mass index (BMI, Kg/m^2^), arterial oxygen tension (PaO_2_, mmHg), EI in acute hospitals (yes, no), NIV in acute hospitals (yes, no), SpO_2_ *change* (%), walking aids requirement (yes, no), and CRP (mg/dL). All analyses were performed using the R statistical software tool (www.r-project.org; version 4.0.5), clustering analyses were performed by the *NbClust* function implemented in the R package called *NbClust*, and 95% CIs of the Spearman correlation coefficient *r* were computed by the *spearman*.ci function implemented in the *RVAideMemoire* package. Functions corresponding to the remaining statistical tests used in the analyses were implemented in the *stats* package. 

## 3. Results

### 3.1. Patients’ Characteristics

Out of the 196 individuals who were admitted during the study period, the data from a total number of 154 participants with complete variable data that could be used for clustering (see Statistical Analysis) were included in the analysis. Demographic and clinical data are shown in Table 1. According to the inclusion criteria, all of participants were normoxaemic at rest. Compared to Pavia, the participants from Lumezzane were more likely to be female, obese, and suffer from congestive heart failure, with a longer LoS in acute hospitals and in study centres and a longer time from admission to acute care hospitals, with a higher Barthel index, CRP, albumin, PaCO_2_, baseline Borg-D, and baseline Borg-F as well as lower D-dimer and ferritin and a lower walked distance at 6MWT and baseline SpO_2_.

### 3.2. Correlation between Clustering Parameters 

As expected, all of participants showed an increase in their HR during the 6MWT, with 81 out of 154 (52.2%) showing EID. Out of 154, 129 (83.8%) and 93 (60.4%) participants showed an increase of at least 0.5 points in Borg-D and Borg-F, respectively at the end-exercise point. 

Measurements of the participants belonging to the different clusters are highlighted with distinct colours and shapes in the scatterplots (grey circles = measures of patients belonging to C1, black crosses = measures of patients belonging to C2). The number reported within each section is the Spearman correlation coefficient describing the strength of the correlation between the variables. As an example, a positive correlation was observed between Borg-D *change* and Borg-F *change* (Spearman r = 0.60); patients belonging to C2 tended to be characterized by higher Borg-D *change* and Borg-F *change* values compared to the patients belonging to C1.

**Figure 1:**
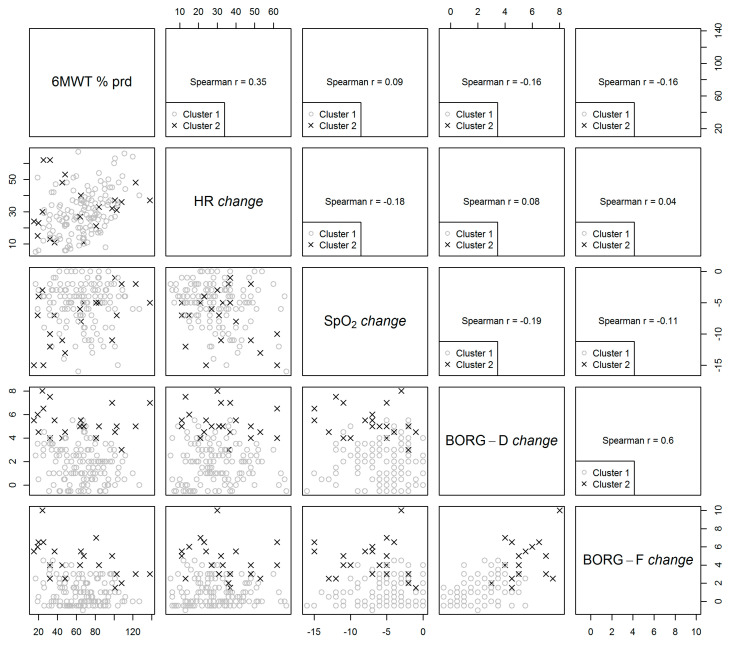
Scatterplot matrix of variables used for clustering analysis.

The scatterplots in Figure 1 describe the correlation between the variables used to identify informative clusters. The Borg-D *change* and Borg-F *change* showed the strongest correlation (r = +0.60, 95% CI = 0.47 to 0.71) followed by HR *change* and 6MWT as well as % predicted (r = +0.35, 95% CI = 0.19 to 0.49). A weaker positive correlation was observed between 6MWT, % predicted, and SpO_2_ *change* (r = 0.09, 95% CI = −0.07 to 0.25), but negative correlations between 6MWT, % predicted, and Borg-D (r = −0.16, 95% CI = −0.32 to 0) and Borg-F *change* (r = −0.16, 95% CI = −0.32 to 0.01) were found. Heart rate *change* was negatively correlated with SpO_2_ *change* (r = −0.18, 95% CI = −0.33 to −0.02), but it was positively correlated with Borg-D (r = 0.08, 95% CI = −0.09 to 0.24) and Borg-F *change* (r = 0.04, 95% CI = −0.12 to 0.20). The SpO_2_ *change* correlated negatively with both Borg-D (r = −0.19, 95% CI = −0.34 to −0.02) and Borg-F *change* (r = 0.11, 95% CI = −0.27 to 0.05).

### 3.3. Clustering Analysis

**Figure 2:**
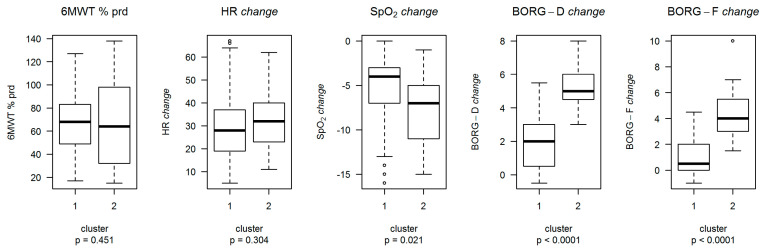
Boxplots describing the distribution of variables used for the analysis by cluster.

Through unsupervised analysis it was possible to identify two clusters: Cluster 1 (C1) included 133 participants (86.4%), and Cluster 2 (C2) included the remaining 21 (13.6%). Compared to C1, the individuals in C2 showed a larger increase in Borg-F and Borg-D and a greater reduction in SpO_2_ (Figure 2 and Table 2).

From top to bottom, each boxplot represented: the lower non-outlier limit, the 25th percentile, the 50th percentile, the 75th percentile, and the upper non-outlier limit. Each point outside of the non-outlier range represents an outlier with respect to the variables’ distribution. *p* = *p*-value deriving from the Wilcoxon rank-sum test.

When focusing on the variables that were not used for cluster definition, we observed that compared to C1, the individuals in C2 were older, more likely to be affected by renal failure and COPD, and had higher baseline Borg-D, *end-exercise* Borg-D, *end-exercise* Borg-F, and lower HR_peak_ (Table 2).

### 3.4. Factors Associated to Variables Used for Clusters Definition

The results of the multivariate feature selection process aimed to identify the most informative subset of factors associated with the set of variables used for cluster definition, which are shown in Table 3 and can be briefly described as follows:

(a) The need of walking aids was associated with lower 6MWT and % predicted. Participants from Pavia were more likely to be characterized by higher 6MWT and % predicted.
(b) History of EI was associated with a positive shift in terms of HR *change* distribution, while SpO_2_ *change*, baseline HR, and time from admission to acute care hospitals were associated with a negative shift in terms of HR *change* distribution. The participants from Pavia were also characterized by a positive shift in terms of HR *change* distribution.
(c) Body mass index was associated with a positive shift in terms of SpO_2_ *change* distribution, and the participants from Pavia centre were also likely to be characterized by a positive shift in terms of SpO_2_ *change* distribution.
(d) Baseline Borg-D and the need of walking aids were associated with a positive shift in Borg-D *change* distribution. SpO_2_ *change* during the test was associated with a negative shift in Borg-D *change* distribution.
(e) The need of walking aids and age were associated with a positive shift in terms of Borg-F *change* distribution.

## 4. Discussion

Survivors of ARF caused by to COVID-19 pneumonia can be distinguished by two clusters that are characterised by different exercise capacities and exercise-induced symptoms. More than 80% of the participants were included in C1, which was characterised by male participants who were able to achieve a better performance during the 6MWT and higher HR_peak_. Older participants with more severe symptoms, greater SpO_2_ *change* during 6MWT, and a greater prevalence of renal failure and COPD were included into C2. The need of walking aids, age, BMI, hospital centre, SpO_2_ *change*, baseline HR, time from admission to acute hospital, history of EI, baseline SpO_2_, and baseline Borg-D were informative with respect to 6MWT, % predicted, changes in SpO_2_, changes in HR, and changes by end exercise-induced severity of dyspnoea and fatigue, respectively.

By cluster analysis, our study has shown the ability to distinguish between the different subgroups COVID-19 pneumonia survivors. Cluster analysis consists of exploratory techniques that are widely used in different research fields, including biology and medicine [10,20]. Clustering allows the discovery subgroups of subjects who share similar characteristics. While our study provides interesting information on exercise capacity in survivors of COVID-19-associated pneumonia, our study is also relevant, as it suggests a modality that can be used to analyse the characteristics of these patients in order to add to the panel of common evaluations. In addition, the characterisation of subgroups according to demographic, clinical, physiological characteristics and response to exercise may have potential clinical usefulness. Different characteristics and responses to a simple and easy to perform exercise test would allow the tailoring of comprehensive management strategies, including exercise training, and may also suggest different hypothetical recovery times after an acute hospital admission or different prognostic consequences in this new population, which is now called post-COVID patients. Prospective studies might confirm the evaluation of these perspectives.

Confirming a previous study [9], our participants showed a wide range of exercise capacities, as assessed by 6MWT values ranging from about 50% to 90%, with median values that were less than 70% of those predicted. To what extent the reduction in exercise capacity was due to the infection and/or to clinical consequences such as prolonged hospital LoS and/or mechanical ventilation is still to be elucidated. The participants in the current study were assessed at a median time of 38 days (min = 8, max = 120 days) from acute hospital admission. However, the time from this event was correlated to change in HR during exercise but not to the distance walked (see Table 3).

In the study by Huang et al. [6], in most individuals who recovered from severe COVID-19, dyspnoea scores and exercise capacity improved over time, but in that study [6], individuals who required EI and mechanical ventilation were excluded due to the potential for the consequences of mechanical ventilation itself influencing the factors that were under investigation. In our study, the need for EI was informative of the changes that take place in terms of HR during exercise but were not indicative of exercise capacity itself (see Table 3). 

Participants from the two centres were different in terms of some demographic, anthropometric, clinical, and physiological characteristics as well as in terms of responses to the exercise test. However, the contributions of the two centres to the clusters did not differ significantly. Of the variables that were characterized by unbalanced distributions between centres, only the baseline Borg-D was differentially distributed between clusters; thus, it is likely that these variables did not heavily influence cluster definition.

### Limitations

As this is a retrospective study, the present work may contain flaws that are associated with such studies, including missing data. Indeed, the pandemic has led to an increase in retrospective publications that have a high level of restrictions [21]. However, we could not wait for well-designed prospective randomized controlled trials before starting interventions in daily clinical practice. Therefore, we feel that with all of the in-built limitations of observational, uncontrolled, or retrospective studies, the scientific community has had to answer the emerging questions posed by the pandemic, which also applied to the field of rehabilitation. Because of this, making timely use of the available data is integral. 

We did not study a control population; however, we are confident that the comparison with predicted 6MWD values allowed us to conduct an unbiased evaluation of the results. 

The lack of lung function assessment may be criticized. This was an unavoidable consequence of the preventive protection measures imposed by the pandemic. Indeed, the study involved patients who were admitted during the biggest outbreak of the pandemic (April–May 2020), when all activities that could potentially spread the infection were suspended, including lung function studies. 

The reported occurrence of commodities in our study must not be considered as a true indication of prevalence, as our patients did not undergo any specific diagnostic tests.

## 5. Conclusions

Despite the limitations of a retrospective design, using cluster analysis, our study allowed us to identify subpopulations of survivors of COVID-19-associated pneumonia. These results might help in the management of the long-term effects of the disease but should be confirmed by prospective, controlled studies.

## Figures and Tables

**Table 1:** Characteristics of studied patients overall and by centre.

Variable	N	Value	Overall	Lumezzane	Pavia	*p*
			N = 154	N = 58	N = 96	
Sex	154	F	45 (29.22%)	23 (39.66%)	22 (22.92%)	0.0298
		M	109 (70.78%)	35 (60.34%)	74 (77.08%)	
Age, years	154		69.5 (60.25:76.00)	70 (61.25:75.75)	68 (59.75:76.25)	0.4432
BMI, Kg/m^2^	154		26.23 (23.46:29.38)	26.64 (23.5:32.24)	26.23 (23.48:28.40)	0.2562
Obesity	154	Yes	37 (24.03%)	22 (37.93%)	15 (15.62%)	0.0024
Arterial hypertension	154	Yes	93 (60.39%)	35 (60.34%)	58 (60.42%)	1
Arrhythmias	154	Yes	25 (16.23%)	10 (17.24%)	15 (15.62%)	0.8197
Diabetes	154	Yes	32 (20.78%)	14 (24.14%)	18 (18.75%)	0.5326
Renal failure	154	Yes	9 (5.84%)	3 (5.17%)	6 (6.25%)	1
Congestive heart failure	154	Yes	14 (9.09%)	11 (18.97%)	3 (3.12%)	0.0024
Asthma	154	Yes	10 (6.49%)	4 (6.90%)	6 (6.25%)	1
COPD	154	Yes	13 (8.44%)	3 (5.17%)	10 (10.42%)	0.3682
OSAS	154	Yes	13 (8.44%)	6 (10.34%)	7 (7.29%)	0.5644
LoS in acute care hospitals, days	154		20 (11:33.8)	26.5 (20:38.5)	16 (10:22)	<0.0001
EI in acute care hospitals	154	Yes	41 (26.62%)	20 (34.48%)	21 (21.88%)	0.0954
Tracheostomy	154	Yes	28 (18.18%)	14 (24.14%)	14 (14.58%)	0.1898
NIV in acute care hospitals	154	Yes	91 (59.09%)	34 (58.62%)	57 (59.38%)	1
O_2_ supply in acute care hospitals	154	Yes	131 (85.06%)	53 (91.38%)	78 (81.25%)	0.0969
LoS in study centres, days	154		17 (13:22)	20 (16.25:28)	15 (12:20)	<0.0001
PaO_2_, mmHg	125		72.6 (65.7:81.3)	71.3 (63.45:79.3)	73.2 (66.5:81.6)	0.2031
PaCO_2_, mmHg	125		35.1 (32.4:38.6)	36.3 (34:39.68)	34.6 (31.5:37.5)	0.0161
pH	125		7.44 (7.42:7.46)	7.44 (7.43:7.45)	7.44 (7.42:7.46)	0.7353
Need of walking aids	153	Yes	47 (30.72%)	15 (26.32%)	32 (33.33%)	0.3757
Motor Barthel index, points	99		90 (80:100)	100 (90:100)	90 (60:100)	0.0018
CRP, mg/dL	144		1.37 (0.68:3.76)	3.4 (1.8:10.2)	1 (0.42:2.06)	<0.0001
D-dimer, μg/mL	121		760 (420:1280)	635 (382.5:1137.5)	920 (465:1460)	0.0378
Ferritin, ng/mL	122		419 (227.75:776.5)	354.5 (213:518)	457 (251:869.5)	0.0300
Albumin, g/dL	105		30.5 (26.9:34.7)	34 (31.1:35.9)	28.3 (25.78:30.83)	<0.0001
Haemoglobin, g/dL	148		11.4 (10.5:12.5)	11.3 (10.7:12.2)	11.5 (10.45:12.65)	0.7520
Time from acute care hospital admission, days	154		38 (29:53)	51.5 (37:71)	35 (26:42.25)	<0.0001
6MWT, m	154		336 (215:446.5)	267.5 (180:372.5)	393 (259.5:464)	<0.0001
6MWT, % predicted	154		67.5 (48:83.75)	53 (37.5:70.5)	73 (56.5:90)	<0.0001
Baseline HR, b/min	154		84 (74:94)	84.5 (75:92)	84 (73.75:94.25)	0.4761
Baseline SpO_2_, %	154		96 (94:98)	94.5 (93:96)	97 (95:98)	<0.0001
Baseline Borg-D	154		0.5 (0:0.5)	0.5 (0.5:0.5)	0 (0:1)	0.0004
Baseline Borg-F	154		0.5 (0:0.5)	0.5 (0.5:0.5)	0 (0:1)	0.0404

Legend: Variable = analysed variable; N = non-missing observations by variable; Value = categorical variables value(s) reported in the table. Data shown as median value of each variable’s distribution (25th:75th percentiles) or absolute and relative frequency (%). *p* = *p*-value from the Wilcoxon rank sum test comparing variables distribution between centres or from the chi-square test for independence between each variable and centre. Abbreviations. BMI = body mass index; Borg D = Borg score for dyspnoea; Borg-F = Borg score for fatigue; COPD = chronic obstructive pulmonary disease; CRP = C-reactive protein; OSAS = obstructive sleep apnoea syndrome; LoS = length of stay; PaO_2_ = arterial oxygen tension; PaCO_2_ = arterial carbon dioxide tension; EI = endotracheal intubation; NIV = non-invasive ventilation; 6MWT = six minutes walking test.

**Table 2:** Results from testing for differences in variables distribution between clusters.

Variable	Value	C1 (*n* = 133)	C2 (*n* = 21)	*p*
Hospital	Lumezzane	50 (37.59%)	8 (38.1%)	1
	Pavia	83 (62.41%)	13 (61.9%)	
LoS in acute care hospitals, days		20 (11:30)	20 (10:39)	0.5737
LoS in study centrrs, days		18 (13:22)	15 (13:23)	0.6221
Time from acute care hospital admission, days		38 (29:52)	39 (25:57)	0.8025
Gender	Females	35 (26.32%)	10 (47.62%)	0.0688
	Males	98 (73.68%)	11 (52.38%)	
Age, years		66 (59:75)	75 (71:77)	0.0081
BMI, Kg/m^2^		26.22 (23.66:29.32)	26.29 (22.49:32.05)	0.7903
Obesity	Yes	31 (23.31%)	6 (28.57%)	0.7879
Hypertension	Yes	81 (60.9%)	12 (57.14%)	0.8099
Arrhytmias	Yes	23 (17.29%)	2 (9.52%)	0.5329
Diabetes	Yes	28 (21.05%)	4 (19.05%)	1
Renal failure	Yes	5 (3.76%)	4 (19.05%)	0.0191
Congestive heart failure	Yes	10 (7.52%)	4 (19.05%)	0.1079
Asthma	Yes	9 (6.77%)	1 (4.76%)	1
COPD	Yes	8 (6.02%)	5 (23.81%)	0.0171
OSAS	Yes	9 (6.77%)	4 (19.05%)	0.0774
PaO_2_, mmHg		73.1 (65.85:81.57)	70.5 (65.25:75.6)	0.2329
PaCO_2_, mmHg		34.95 (32.32:38.55)	36.2 (33.6:39.5)	0.3233
pH		7.44 (7.42:7.46)	7.43 (7.42:7.46)	0.8998
Endotracheal Intubation	Yes	39 (29.32%)	2 (9.52%)	0.0640
Tracheostomy	Yes	26 (19.55%)	2 (9.52%)	0.3638
NIV	Yes	81 (60.9%)	10 (47.62%)	0.3429
O_2_ supply	Yes	115 (86.47%)	16 (76.19%)	0.3147
Need of walking aids	Yes	38 (28.79%)	9 (42.86%)	0.2132
Motor Barthel index, points		95 (83.75:100)	90 (65:100)	0.2150
CRP, mg/dL		1.39 (0.71:3.54)	1 (0.51:6.67)	0.8274
D-dimer, μg/mL		750 (420:1270)	1125 (522.5:1440)	0.2634
Ferritin, ng/mL		434 (231.5:794.5)	356 (198.75:440.25)	0.2000
Albumin, g/dL		30.6 (26.9:34.6)	29.35 (26.85:35.38)	0.9431
Haemoglobin, g/dL		11.5 (10.55:12.55)	11.2 (10.5:11.7)	0.2728
6MWT, m		355 (225:450)	267 (150:372)	0.0501
6MWT, % predicted #		68 (49:83)	64 (32:98)	0.4514
Baseline HR, b/mim		84 (74:94)	84 (69:92)	0.4754
HR_peak_, b/min		154 (145:161)	145 (143:149)	0.0084
HR, % of maximal predicted		74 (66:82)	76 (72:81)	0.2380
HR *change*, % #		28 (19:37)	32 (23:40)	0.3044
Baseline SpO_2_, %		96 (94:98)	96 (95:97)	0.7778
End exercise SpO_2_, %		91 (88:94)	89 (87:91)	0.0806
SpO_2_ *change*, % #		−4 (−7:−3)	−7 (−11:−5)	0.0206
Baseline Borg-D		0.5 (0:0.5)	0.5 (0.5:2)	0.0291
End exercise Borg-D		2 (1:3)	6 (5:7)	<0.0001
Borg-D *change* #		2 (0.5:3)	5 (4.5:6)	<0.0001
Baseline Borg-F		0.5 (0:0.5)	0.5 (0:0.5)	0.7279
End exercise Borg-F		1 (0:3)	5 (4:7)	<0.0001
Borg-F *change* #		0.5 (0:2)	4 (3:5.5)	<0.0001

Legend: Variable = analysed variable; Value = categorical variables value(s) reported in the table; C1 = median (25th:75th percentiles) or absolute and relative frequency (%) of each variable’s distribution in cluster 1; C2 = median (25th:75th percentiles) or absolute and relative frequency (%) of each variable’s distribution in cluster 2; *p* = *p*-value from the Pearson chi-square test for independence. # Variable used for clusters definition. Abbreviations. 6MWT: Six-minute walking distance test; BMI: body mass index; COPD: chronic obstructive pulmonary disease; CRP: C-reactive protein; HR: heart rate; NIV: non-invasive ventilation; OSAS: obstructive sleep apnoea syndrome; SpO_2_ pulse oxygenation.

**Table 3:** Variables informative with respect to 6MWT % pred, HR % *change*, SpO_2_ *change*, Borg-D *change*, and Borg-F *change*.

	**6MWT (% Predicted)**	
**Variable**	**Coefficient (95% CI)**	***p* value**
Intercept	61.92 (56.24:67.60)	
Hospital = Pavia ^#^	20.03 (13.21:26.85)	<0.0001
IWalking Aids = Yes	−27.28 (−34.45:−20.10)	<0.0001
	**HR *change***	
**Variable**	**Coefficient (95% CI)**	***p* value**
Intercept	54.97 (40.04:69.9)	
Hospital = Pavia ^#^	5.37 (0.71:10.04)	0.0243
Baseline HR, b/m ^#^	−0.33 (−0.48:−0.18)	<0.0001
SpO_2_ *change*	−1.12 (−1.67:−0.57)	<0.0001
Time from hospital admission, days	−0.19 (−0.29:−0.08)	0.0009
EI = Yes	5.84 (0.98:10.71)	0.0189
	**SpO_2_ *change***	
**Variable**	**Coefficient (95% CI)**	***p* value**
Intercept	−35.21 (−59.74:−10.69)	
Hospital = Pavia ^#^	1.42 (0.2:2.65)	0.0228
Baseline SpO_2_, % ^#^	0.25 (−0.01:0.5)	0.0603
BMI	0.2 (0.1:0.3)	<0.0001
	**Borg-D *change***	
**Variable**	**Coefficient (95% CI)**	***p* value**
Intercept	0.67 (−0.09:1.42)	
Hospital = Pavia ^#^	0.59 (−0.02:1.2)	0.0591
Baseline Borg-D ^#^	0.48 (0.04:0.92)	0.0317
Walking Aids = Yes	1.01 (0.38:1.64)	0.0018
SpO_2_ *change*	−0.13 (−0.21:−0.04)	0.0027
	**Borg-F *change***	
**Variable**	**Coefficient (95% CI)**	***p* value**
Intercept	−1.39 (−3.30:0.53)	
Hospital = Pavia ^#^	0.17 (−0.44:0.78)	0.5844
Baseline Borg-F ^#^	0 (−0.35:0.35)	0.9971
Walking Aids = Yes	1.10 (0.45:1.75)	0.0010
Age	0.04 (0.01:0.06)	0.0112

Legend: Variable = variable selected by stepwise linear regression; Coefficient (95% CI) = linear regression coefficient and corresponding 95% confidence interval; *p* = *p*-value. Intercept indicates the baseline coefficient. ^#^ Variable forced to be included in the model. Regression coefficients can be interpreted as in the following example: patients from Pavia hospital are characterized by a shift in terms of mean value of the SpO_2_ *change* distribution of +1.42%, holding the other variables included in the model (BMI and baseline SpO_2_) constant. Abbreviations: 6MWT: Six-minute walking distance test; BMI: body mass Index; EI: endotracheal intubation; HR: heart rate, SpO_2_: pulse oxygenation.

## Data Availability

Anonymized data materials will be made publicly available at https://www.zenodo.org/.
